# How do adolescents with cerebral palsy participate? Learning from their personal experiences

**DOI:** 10.1111/hex.12796

**Published:** 2018-06-01

**Authors:** Sophie Catharina Wintels, Dirk‐Wouter Smits, Floryt van Wesel, Johannes Verheijden, Marjolijn Ketelaar, the PERRIN PiP Study Group, Anna van der Leest, Coosje de Groot, Dion Snel, Jesse van de Water, Lauren Sluiter, Nando de Bruijn, Nathan Janssen, Nienke Heijne Makkreel, Nikita Kramer, Piotr Bouma, Sam Vergeer, Thom den Boer, J.M. Voorman, A.J. Dallmeijer, M.E. Roebroeck, H.A. Reinders‐Messelink, J.W. Gorter

**Affiliations:** ^1^ Center of Excellence for Rehabilitation Medicine Brain Center Rudolf Magnus University Medical Center Utrecht University Utrecht and De Hoogstraat Rehabilitation Utrecht The Netherlands; ^2^ Department of Methodology & Statistics Utrecht University Utrecht The Netherlands; ^3^ BOSK Association of Physically Disabled Persons Utrecht The Netherlands

**Keywords:** adolescents, cerebral palsy, participation, patient involvement, personal experiences

## Abstract

**Background:**

Participation in society can be difficult for adolescents with cerebral palsy (CP). Information is often based on quantitative studies, and little is known about their personal participation experiences.

**Objective:**

The aim of this study was to examine the participation experiences of adolescents (aged 12‐17 years) with CP.

**Methods:**

A qualitative participatory research method was used. Twenty‐three semi‐structured open interviews were conducted with 13 male and 10 female adolescents (mean age 15 years) with CP. An interview checklist was developed jointly with adolescents with CP. This checklist ensured that the adolescents reflected on various participation areas, that is school, sports, health care and work. The analysis was based on principles of grounded theory.

**Findings:**

From the adolescents’ experiences, 4 key categories were identified. One concerned participation, as such, expressed as “My participation experiences,” including experiences, thoughts and feelings while participating in daily life. Three other categories concerned factors that influence participation experiences, expressed as “My disability,” “Me as a person” and “My environment.” These 4 categories together formed a model showing the interactions and dynamics of participation according to adolescents with CP.

**Conclusion:**

Adolescents with CP expressed their participation experiences, including various important influencing factors. This study conceptualized these experiences into a dynamic model. This experience‐based participation model may provide new, personalized perspectives for practice, for instance in rehabilitation, but also for schools and sports (or sports clubs) attended by adolescents. Focusing on personal and environmental factors might be the key to successful participation.

## 1 INTRODUCTION

Cerebral palsy (CP) is one of the most common childhood disabilities.^1^ Children with CP have permanent disorders of movement and posture, which limit their activity, and are often accompanied by disturbances of sensation, perception, cognition, communication and behaviour, as well as by epilepsy and secondary musculoskeletal problems.^2^ CP can thus affect the development of daily functioning throughout the lifespan.^3^ Consequently, participating in society with others on an equal basis can be difficult for children with CP.

The International Classification of Functioning, Disability and Health, Child and Youth version (ICF‐CY) describes participation as “involvement in a life situation.” Participation is seen as a dynamic interaction between a person and his/her environment.^4^ As children develop, their life situation and social environment change and become more complex. Adolescents have to cope with physiological and emotional changes and are confronted with social transition.^5^ This critical transition period can be particularly difficult for adolescents with a disability, such as CP. They are confronted with disability‐related challenges during transitions.^6^, ^7^ For example, they may depend on their parents’ assistance for personal care, while having a desire to become more autonomous.^6^ Another example is that they may have to cope with negative comments from others.^7^ How do adolescents with CP manage to participate during this transition period?

Most studies regarding the participation of persons with CP seem to have focused on the childhood period in general.^8^, ^9^, ^10^ Fewer studies have focused specifically on adolescence. Donkervoort et al^11^ showed that 20%‐30% of adolescents with CP aged 16‐20 years faced restrictions in daily life and social participation. Livingston et al^12^ confirmed these findings and found that leisure activities, mobility, school and socialization were issues mentioned most frequently by adolescents with CP and their caregivers. Engel‐Yeger et al found that adolescents aged 12‐16 years with CP engaged in a smaller range of activities outside of school than typically developing adolescents. Compared to adolescents without CP, they performed these activities less frequently, more often alone and at home.^13^ The European study by Michelsen et al^14^, involving a large group of adolescents with and without CP, confirmed these results. They found that adolescents with CP spent less time with friends and had less autonomy in their daily lives. From these studies, we can conclude that participation is indeed difficult and not self‐evident for adolescents with CP.

The above‐mentioned studies focused on the numbers of activities and restrictions for these adolescents and gave little insight into how adolescents experience their involvement in life situations. This conceptual distinction is consistent with the views of Imms et al^15^, who described that participation consists of *attendance* to certain activities, but also *involvement* (thoughts, feelings, personal stories, etc.) when doing so. In most studies published so far, the stories behind the numbers are lacking. This is a serious shortcoming, as, while growing older and making the transition into adulthood, adolescents develop their own ideas and experiences. If we want to understand their thoughts and feelings when being involved (or not) in daily life situations, we must ask the adolescents themselves about their personal experiences.

Although limited, several studies have described personal participation experiences (ie “involvement”) of adolescents with CP. These studies appear to have focused mainly on sports. For instance, Shimmel et al and Verschuren et al conducted interviews and focus group sessions with children and adolescents with CP and their parents regarding their participation in physical activities (PA). These studies identified physical limitations, environmental and personal factors that influenced PA. For example, participants in both studies reported that accessibility, social support and self‐perception affected their participation in PA.^16^, ^17^ A review by Lindsay presented an overview of the experiences of children and adolescents with CP regarding their CP in a broader context than only sports/PA. Their results show that the experiences of children and adolescents with CP aged 2‐25 years focused on social inclusion, physical environment, the role of family and peers and participation. Although experiencing physical impairments (and the consequences thereof) and social exclusion, adolescents with CP used strategies to cope with their CP, like keeping up their motivation and adapting to situations.^18^ In accordance with the description of participation in ICF‐CY^4^, these studies show that—from a subjective experiential perspective—the environment, but also personal factors, plays an important role in enabling participation.

These studies concentrated on children with CP in a wide age range. Adolescents, however, are in a stage of transition and have to deal with a changing involvement in life situations. Whether someone perceives factors to be supportive or limiting also changes between childhood and adolescence, as Shimmel et al^16^ emphasize. This would seem to call for a narrower focus on adolescents, not only regarding the age range of the study sample but also by letting their own perspective be the guiding principle; not talking *about* them, but *with* them. The main purpose of this study was to examine the personal participation experiences of adolescents (aged 12‐17 years) with CP. To capture the full scope of their potential participation, we asked adolescents with CP to share their participation experiences in several daily life areas: school, sports, health care and work.

## 2 METHODS

### 2.1 Design

This was a qualitative study, using a participatory research design, and was a 10‐year follow‐up study of children with CP who were engaged in PERRIN, a large longitudinal research programme in the Netherlands.^19^, ^20^

### 2.2 Experiential experts

To understand the experiences of adolescents, we asked them to be participants not only in the sense of study subjects. A group of 12 adolescents with CP participated as “ambassadors,” involving them actively as experiential experts in all stages of the project. The ambassadors were selected in co‐operation with BOSK (Dutch Association of Physically Disabled Persons), whose team members also assisted the ambassadors during the process. Ambassadors gave input regarding the study protocol and the form and content of the interviews and were involved in analysis and member check.

### 2.3 Participants

**Figure 1:**
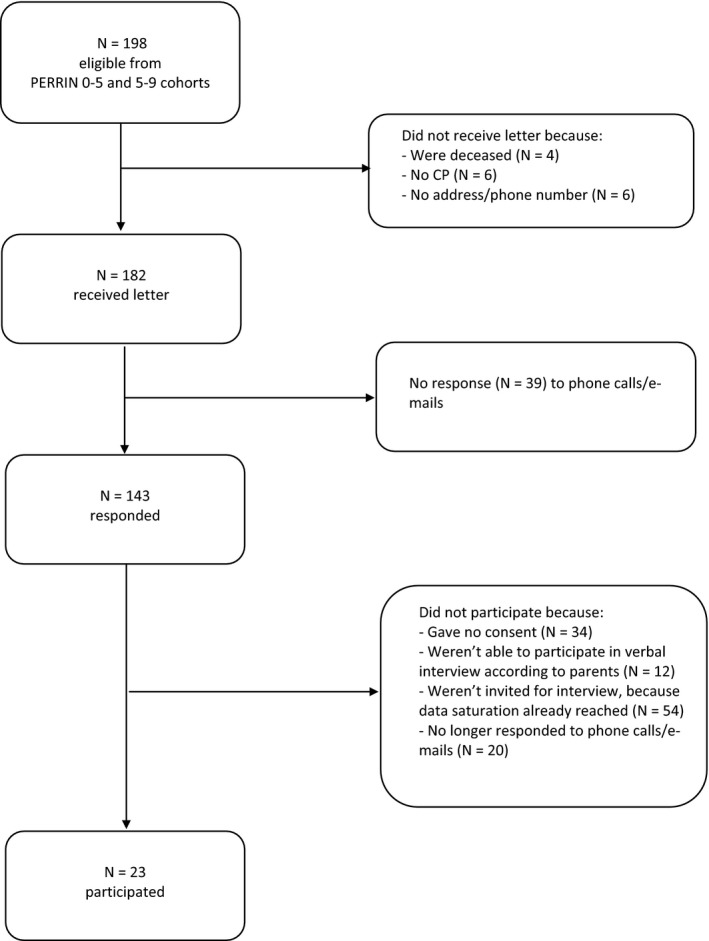
Sample selection and recruitment

Eligible for participation were persons diagnosed with CP from existing PERRIN cohorts that started when they were 2‐7 years old.^19^, ^20^ Ten years after their first recruitment in PERRIN, so at the age of 12‐17 years, participants were recruited again for this study. Information letters, accompanied by an introductory letter from the relevant doctor, were sent to the adolescents and their parents. After at least 2 weeks, the adolescents and their parents were telephoned to enquire whether they were interested in participating. To be included, participants had to be able to take part in a verbal interview (which was decided in consultation with the parents). Figure 1 shows a flow chart of the sample selection and recruitment process.

A total of 23 adolescents, 13 male and ten female, with a mean age of 15 years 3 months (SD 2 years 3 months) participated. Table 1 shows participants’ characteristics. People with CP are often characterized by their level of gross motor functioning, measured with the Gross Motor Classification System (GMFCS),^21^ and their level of communication, measured with the Communication Function Classification System (CFCS).^22^ The GMFCS and CFCS scores both range from level I to V, increasing with the severity of the motor or communication disabilities. In this study, all levels were represented, except for CFCS level V.

**Table 1:** Characteristics

Age, mean (SD)	Years, months	15, 3 (2, 3)
Age, N (%)	12 years	3 (13.0)
	13 years	1 (4.3)
	14 years	8 (34.8)
	15 years	5 (21.7)
	16 years	2 (8.7)
	17 years	4 (17.4)
Gender, N (%)	Male	13
	Female	10
GMFCS (%)	Level I	14 (60.9)
	Level II	5 (21.7)
	Level III	2 (8.7)
	Level IV	1 (4.3)
	Level V	1 (4.3)
CFCS (%)	Level I	15 (65.2)
	Level II	4 (17.4)
	Level III	2 (8.7)
	Level IV	2 (8.7)

CFCS, Communication Function Classification System; GMFCS, Gross Motor Function Classification System; SD, standard deviation; N, number.

### 2.4 Measurements

Semi‐structured open interviews were held, using an interview checklist. The checklist was developed by the authors, based on input from the above‐mentioned ambassadors (adolescents with CP collaborating in the project). After 2 interviews had been held, the interview checklist was evaluated by the authors and ambassadors and was found to provide sufficient information on participation experiences. The interview involved questions and a set of word cards in a box (see Appendix A). The interviewer started with an open question. Depending on the answers given by the participants, the interviewer then probed them to share their experiences. The word cards were used as a fun and active way to encourage respondents to share more experiences and to increase their involvement.^23^

### 2.5 Procedure

Interviews were carried out between March and November 2016. The majority of the interviews (N = 21) were conducted by the first author of this article, while 2 interviews were conducted by the second author. Both authors had been trained in conducting interviews and were familiar with people with CP. The interviews took place at participants’ homes and lasted 48 minutes on average (ranging from 20 to 89 minutes). All interviews were voice‐recorded and archived within a protected digital environment and were transcribed verbatim. Verbatim transcripts were numbered and did not contain immediately identifiable information, such as names.

### 2.6 Ethical considerations

The adolescents who participated, and their parents or legal representatives, gave their written informed consent before the interviews were conducted. Participants could discontinue the interview at any time without giving a reason. Before the interview, the adolescents agreed to the interview being audio‐recorded and gave their verbal agreement again on tape. The medical ethics committee of the UMC Utrecht judged that this study (protocol no. 15‐669/C) did not fall under the scope of the Dutch Medical Research Involving Human Subjects Act (WMO). The study was also approved by the internal scientific committee of De Hoogstraat Rehabilitation.

### 2.7 Analysis

The MAXQDA software program, version 12,^24^ was used for qualitative data analysis. The transcripts were analysed according to the open, axial and selective coding procedure described by Boeije.^25^ This procedure is based on principles from grounded theory as described by Corbin and Strauss.^26^ Principles of constant comparison and theoretical sensitivity were applied during the process of analysis^25^, ^26^.

In the first stage, *open coding and axial coding* were applied iteratively*,* with the first and second authors working closely together. They independently coded 4 interviews. Two of these interviews were also coded by 2 ambassadors in one‐on‐one meetings with the first author. Both authors discussed similarities and differences in coding until agreement was reached. After several meetings of reviewing and fine‐tuning, a code tree was formed. All codes assigned by the ambassadors, accompanied by their comments and insights, were included in this process. The first author coded the remaining interviews, 2 of which were also coded again by 2 ambassadors. Whenever new codes or categories emerged, these were discussed with the second author and the code tree was modified. The result of this stage was a definitive code tree in which all codes were clearly defined. In the second stage, *selective coding*, the identified codes were discussed within the context by both authors. Codes were merged into categories consisting of different themes. A preliminary thematic model was designed by the first and second authors and was presented to the other authors and discussed, which led to an even more fine‐tuned thematic model.

### 2.8 Member check

The thematic model with categories and themes that arose from the interviews were presented to and discussed with 3 of the ambassadors, 2 of whom were also interviewed as participants in this study. The ambassadors regarded the findings as recognizable and appropriate to their own situation and that of adolescents with CP in general. Following the member check, no further changes to the findings were made.

## 3 FINDINGS

Qualitative analysis of the interviews showed that the experiences of the respondents fell under 4 main categories: one regarding participation as such (i) and 3 regarding factors that influence(d) their participation (ii, iii and iv). We called these (i) My participation experiences, (ii) My disability, (iii) Me as a person and (iv) My environment (a. My social environment, b. My physical environment).

**Figure 2:**
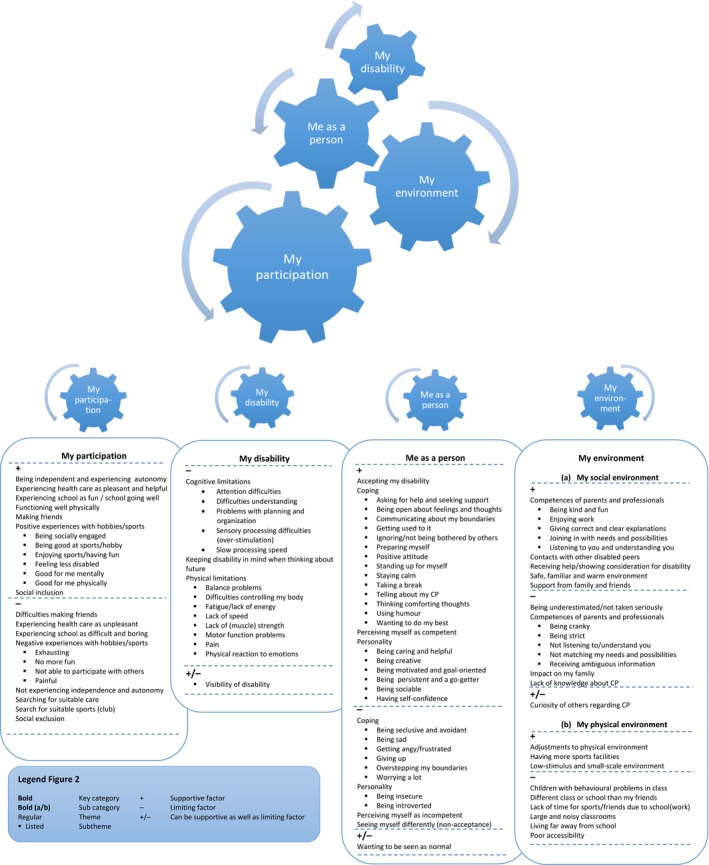
Participation model

These categories and related themes cannot be interpreted separately, as they are intertwined and as such form a dynamic participation model (Figure 2). Factors regarding adolescents’ disability, but also psycho‐social factors such as the environment and their personality affect participation experiences and vice versa. The model shows how the categories and underlying themes are related.

All categories, with themes and subthemes (in *italics*), are shown in the model and are explained below, supported by participants’ quotes.

### 3.1 My participation experiences

The respondents spoke about their participation or non‐participation in several areas of life. They mentioned attending health care, school, sports and (future) work. The themes found to be related to their experiences, thoughts and feelings are described below.

When discussing the topic of health care, the experiences were varied. Respondents were familiar with rehabilitation centres, hospitals and various kinds of therapy, but also with the use of different assistive devices. They found it difficult to find the appropriate (health)care (*Searching for suitable care*), especially the right orthopaedic shoes. On the one hand, various forms of care were *experienced as unpleasant*, but, on the other hand, they were also *experienced as pleasant and helpful*. One girl with CP told how a baclofen pump helped her to participate more:

Yes, I don’t know how to put it… It’s just that [because of the Baclofen pump] I’m less stiff and have less pain everywhere, in my knees and such and everywhere. I can sit better too and do more things. Cycling and stuff. (girl, 17 years, using an electric wheelchair)

One 17‐year‐old girl also told how she loved being creative, as that was one of the few things she could do by herself without needing help. *Being independent and experiencing autonomy* was also a theme for other adolescents and related to different areas in their lives (health care, school, sports and work). Besides experiencing independence and autonomy, respondents described how they *not experienced independence and autonomy* or had the feeling that others decided things for them. One 15‐year‐old boy, who attended special education, told about his experience with therapy and felt there was little room for his opinion:

It was agreed with the rehab doctor that the occupational therapist (OT) would follow me regarding the flexibility of my hands. I don’t agree you know. No. But my mum made me. I have the idea that it’s not very useful […] And what I’m also worried about: they throw me in. You’ve got hours in which you do your homework. But those are also the hours for OT, speech therapy, physiotherapy. And I can’t stand it. I can’t stand that I am doing my homework and then have to leave. (boy, 15 years, feels at home at his school and is a fanatical field hockey player)

Respondents described how they enjoyed school, *experiencing school as fun or doing well at school*. However, negative experiences were also mentioned by the respondents, some stating that they *experienced* s*chool as boring or found school difficult*. These negative experiences with school were sometimes caused by *social exclusion*, which was a theme mentioned regarding all daily life areas. They described being socially excluded and having *difficulties making friends*:

Er yeah, and all in all I find that I’m being looked down upon because I walk differently. Ah well, it is just difficult to make contact with other kids. And er, they usually have their own friends, but, er, yeah I just feel, I feel I don’t fit in. (boy, 15 years, loves cycling, often feels depressed because of his CP and social exclusion)

Although being socially excluded at times, respondents also described *making friends* and being *socially included*:

For instance, when I can’t keep up they [friends] will help me without getting impatient or something. That means they don’t mind if I sometimes, that I’m dreaming or making odd movements, or that I have trouble at gym or things like that. Yeah, they just help me and then, yeah, … they do accept me the way I am. (girl, 12 years, has attention difficulties, is fond of drawing and gaming)

Regarding sports and hobbies, all respondents reported *experiencing their hobbies and sports favourably* and described several reasons for this. For example, this 17‐year‐old girl explained how horse riding made her *feel less disabled*:

I hugely enjoy sitting on a horse. Because I don’t feel disabled then. Well, I feel, er, yeah, I just sit up straight. And they… er, they used to say you can’t sit up straight on a horse and can’t ride a horse without someone holding on to you. […] And… I can even trot on my own. I used to have someone next to me to keep my balance. But I can do that by myself now. (girl, 17 years, wheelchair‐bound, her passion is horseback riding)

Some respondents *functioned well physically* and were able to participate. For example, one male participant stated that he loved gym class and was able to participate—even when wearing his splints. By contrast, the respondents experienced difficulties finding the right sport/sports club (*search for suitable sports*). Some respondents had tried several sports or clubs in the past, but stopped practicing sports as they were *not able to participate with others*, found it *no more fun* and/or experienced sports as *painful* or *exhausting*. One boy described how he tried playing soccer, but was not able to participate with team members:

That [soccer] was fun all‐right, but I often didn’t get the ball. Because I wasn’t fast enough. I didn’t like that too much. I quit because I didn’t like it all that much anymore. Mostly because of that. (boy, 17 years, who likes playing sports and whose favourite class is gym class)

This quote also shows how physical limitations (ie *lack of speed*; see My disability)—resulted in this boy with CP being *socially excluded* during soccer.

### 3.2 My disability

Respondents reported several factors related to their disability that influenced their participation. They mentioned *cognitive* and *physical limitations*. As regards physical limitations, respondents described suffering from *fatigue* and had limited energy, which influenced their ability to participate like adolescents without CP. For one girl, this was the reason why she went to school by bus instead of bicycle:

I don’t go to school on a pedal bike. I go to school by bus because when I get to school I am just too knackered and then the day hasn’t even started yet. And when school is done, right, I am very tired as well and then I will go, I take the bus home. (girl, 14 years, finds it difficult to talk about her CP with classmates)

Another important aspect appeared to be the *visibility of their disability*, especially in interactions with their environment. As referred to above, a 15‐year‐old boy described how it was difficult to make friends. He also mentioned that this was partly due to the visibility of his CP (“walking differently”) which made him feel that others looked down on him, and which prevented him from getting in touch with other children.

When thinking about their future, respondents did *keep their disability in mind*, as well as the limitations they experienced. They thought about the amount of care they will need in the future and what influence their disability might have on their future work situation (eg not being able to drive a truck due to physical limitations).

### 3.3 Me as a person

Multiple factors regarding adolescents “as a person” were identified that influenced the respondents’ participation experiences. These personal factors were apparent within a broad range of contexts.

Several *personality* traits were identified that influenced the respondents’ participation experiences, with positive and supportive personality traits being mentioned most. One 14‐year‐old girl described how *having (more) self‐confidence* thanks to her girlfriends made her participate better in sports over the years:

In the past, I wasn’t able to do certain things in gym. For example, jumping over a vaulting box. But now I can do that and can also do other things. I think because I got more self‐confidence. I dare to do more and I find that I’m capable of doing much more than I used to think. […] I think it’s mainly because of my friends that I got more self‐confidence. They stick up for me. (girl, 14 years, loves to dance)

The way in which participants perceived themselves and their disability (*accepting my disability* and *perceiving myself as competent*/*incompetent)* was important for the way respondents experienced life situations and were able to participate successfully. Their thoughts and ideas showed their need to be “normal” and to be seen as such (*Want to be (seen as) normal*). Respondents’ participation experiences influenced the way in which they perceived themselves as well as their disability. As the quote by one 17‐year‐old girl shows, not being able to participate because of her CP and perceiving incompetence influenced how she felt about her disability:

I haven’t really accepted my disability. […] Because there are certain things that I can’t do. That’s what it comes down to. I think: I want it, but I can’t. […] Now I’m getting to an age where I start to think that I don’t want this disability. You start to notice that there are certain things that you’re not able to do. I used to think I would learn to do those things when I’d get older, but no. I just can’t do them anymore. (girl, 17 years)

Different ways of *coping* were mentioned in the interviews. Respondents used a broad range of strategies to cope with daily life situations. Suitable strategies like *asking for help and seeking support* were mentioned, as well as less suitable strategies such as *being seclusive and avoiding contacts*.

### 3.4 My environment

Participants described environmental factors that affected their participation. Within this key category, 2 subcategories were identified: (i) My social environment and (ii) My physical environment.

#### 3.4.1 My social environment

The social environment was experienced to be supportive as well as limiting as regards respondents’ participation in all the different areas. Respondents experienced practical and emotional support from their social environment. Some respondents described having *contacts with other disabled peers*, which made them feel like they were “not the only one” with a disability. Furthermore, most respondents received *support from family and friends*, for example, helping with activities of daily living.

Receiving help from their family also had a downside, as some respondents mentioned, referring to the *impact of their disability on their family*:

Well, I find my family also have to adapt to my condition. […] Yeah, my mum always needed to come with me to go there, or my dad, and then it was like: who will go this time, what’s easiest with regard to work. That kind of thing. (girl, 14 years, enjoyed visiting the rehabilitation centre because her mum helped make it a fun day)

Respondents mentioned several *competences of their parents and professionals* (ie health‐care professionals, teachers and trainers) that influenced their participation, for example, *listening to you and understanding you* as supportive of their participation, as opposed to *not listening to you and understanding you*, which was a limiting factor.

In general, a *safe, familiar and warm environment* and *receiving help and showing consideration for my disability* were experienced as pleasant by the respondents and encouraged them to take part in sports, be successful at school and have a say in decisions about their health care. Although other people seemed *curious about their disability*, a *lack of knowledge about CP* and *being underestimated or not taken seriously* by others were experienced as unpleasant and not supportive of their participation. One of the 14‐year‐old boys with CP told how he was not taken seriously by his coaches:

Well for instance at hockey I only counted as half a man because I can’t keep up that long. Well, my… I first had two coaches, but they quit. So we got a new coach. It was these two previous coaches who said that. I wasn’t really happy [that they said that]. (boy, 14 years, has difficulties running but likes playing sports with peers)

#### 3.4.2 My physical environment

In their physical environment at school and at sports, the respondents experienced *low‐stimulus and small‐scale environments* as supportive, as they offered more peace and space for them to focus and get personal attention. In school, *large and noisy classrooms* and the presence of *children with behavioural problems in their class* prevented them from focusing on their schoolwork and made it more difficult for them to become socially included. Also, being in a *different class or school than friends*,* lack of time for sports and friends due to lessons or school work*,* living far away from school* and *poor accessibility* were experienced as limiting their participation inside and outside of school. Poor accessibility to a swimming pool made this 17‐year‐old boy quit swimming:

I quit at a certain point. In fact because, er, it just became too strenuous for me really, because after swimming you have to get out of the water of course. And they really had only one little stairs that went straight up. And that became more and more tiring, up to the point where it just didn’t work out at all. (boy, 17 years)

However, various *adjustments to their physical environment* and *having more sports facilities* made it possible for the respondents to participate more fully in school, sports, health care or work. For example, a 17‐year‐old girl described how an adjustment to her environment made her less tired during work:

I stood at the leftmost cash desk and then you could more easily reach everything so to speak, so you didn’t have to walk too far or whatever. My employer arranged that for me, no problem. (girl, 17 years)

## 4 DISCUSSION

This study examined personal experiences of adolescents with CP regarding participation in several daily life areas. Their participation experiences were diverse, and the interviews yielded rich information. Besides sharing their participation experiences, adolescents expressed what limited and what supported their participation in several life areas. In the present study, we conceptualized these experiences and limiting/supportive factors into a dynamic model.

Our results show that adolescents with CP do have difficulties participating, and participation on an equal basis with others is an issue. However, their experiences are not all “CP‐specific.” From this, we would like to emphasize that adolescents with CP are first of all adolescents with the same hopes, dreams, thoughts and feelings as every adolescent.

In addition, the results show that adolescents with CP are able to cope with daily life situations, including participation difficulties. Our respondents emphasized the importance of personal and environmental factors in overcoming these difficulties. The ICF(‐CY) acknowledges the relation between environmental and personal factors and participation, but these factors are not specified in detail in the ICF. Compared to some earlier studies^16^, ^17^, ^18^, we were able to further elaborate on these factors and investigate them more thoroughly. Supported by their environment and personality, adolescents with CP can overcome challenges, whether directly related to their disability or not.

The adolescents we interviewed mentioned factors relating to their CP that limited their participation, which corresponded to those reported by previous studies^11^, ^12^, ^16^, ^17^, ^18^. Physical factors have been addressed in rehabilitation for many years,^27^, ^28^ and neurological and cognitive limitations regarding CP are also receiving more and more attention in current rehabilitation practice.^29^ However, what our study adds is that the visibility of the disability seems to play an important role for adolescents when participating in daily life. This might be because adolescents with CP, as this study’s respondents mentioned, have the need to be seen as normal. Visibility and the desire of adolescents with CP to be normal is something professionals in rehabilitation, school, sports or work or anyone in “their environment,” should be sensitive to.

A strength of this study is that we engaged adolescents with CP in all aspects of the study. Not only did we ask the adolescents themselves about their experiences, we also involved 12 adolescents with CP, who were experiential experts, as ambassadors in our project. They were of great value in setting up and conducting our research, for example, when deciding upon the best way to interview other adolescents. As researchers, we thought photo‐elicitation would be a good method of interviewing, the ambassadors said that they would be more comfortable with simply being asked questions and using the set of word cards for the first conversation with someone they do not know. Also, their reflections on other adolescents′ experiences helped us choose the right labels for categories and themes in our analysis and confirmed our findings in the member check.

One limitation of this study is that adolescents with GMFCS level I were overrepresented. More severe levels of motor dysfunction are associated with greater intellectual disability and/or communication problems.^30^ This might explain the overrepresentation, as the participants had to be able to take part in an interview. However, the respondents with level II‐V who participated had similar participation experiences as those with level I. Still, we would recommend that future research focus more specifically on severe levels of CP as well. Understanding the experiences of adolescents with severe intellectual and/or physical disabilities requires other research methods, for example, by proxy, observation or physiological methods.^31^, ^32^

The experience‐based participation model presented in this study may provide new perspectives for practice, such as in the field of (paediatric) rehabilitation, but also for schools, sports clubs and work which adolescents engage in. The adolescents with CP in our study were able to overcome disability‐ and non‐disability‐related challenges, but were dependent on their environment and personality in doing so. However, we still seem to focus too often on disability‐related factors. Environmental and personal factors can be changed and might provide new perspectives for practice. Little research has been carried out into interventions focusing on personal and environmental factors^28^, even though these factors might be the key to successful participation.

The adolescents in our study reflected on factors that supported and limited their participation in several daily life areas. Our ambassadors reflected on the experiences of other adolescents as well, which shows their ability to contribute to discussions about the participation of adolescents. The ambassadors in this study absolutely created added value to this research, and we want to emphasize the importance of co‐creation in research: involve them not only as participants, but also as contributors.

## CONFLICT OF INTEREST

The authors have no conflict of interests.

## APPENDIX A Interview checklist

### A.1

**Table:**

Introduction	
□ Introduce myself	I am [NAME]I am a junior researcher
□ Introduction to interview	Research on adolescents with cerebral palsyConversation/’interview’ about participationYour personal experiences: no wrong answers
□ Consent for audio‐taping	Recording for later playback, transcription and analysisIs that okay for you?I will ask you again during taping so we have your consent on tape
□ Questions?	Do you have any questions?

**Table:**

Starters
□ Can you tell me something about yourself?*When nothing follows:*□ What does a week look like for you?□ What do you think of when you hear the word ‘participation’? How is that for you?

**Table:**

Topics	
□ School□ Sports□ (Health)care□ Work	Can you tell me something about…?How is participation in…?
	*Additional questions*What school do you visit? How are things at school?Which sport do you practice? What’s that like?Do you get any health care? What kind of care? How do you feel about that care?Do you have a job? What’s it like at work?
□ Positive experiences	What goes well?What do you like about participating in…?
□ Negative experiences	What does not go so well?What could be better?What do you dislike most?
□ Past	What was that like in the past (when you were a child)?What was that like at primary school?
□ Present	What is that like now (as an adolescent)?What is that like now in secondary school?
□ Future	What are your dreams for the future? [*optional question]*What do you think that will be like in the future?Do you have an idea of what that will be like in the future?What are your expectations for the future (when you are an adult)?

**Table:**

General additional questions	
What do you mean? Can you explain that?Can you give an example?Can you tell me more about it?Why?What’s that like for you?What is your way of dealing with it?Can you keep up with others?Are there any adjustments or assistive devices? Which ones?	Do you have any tips for…?Do you have questions about…?How can health care professionals/teachers/trainers approach you best?How do you make sure you can keep up? What do you need for that?What helped you with this?

**Table:**

Word cards in box		
This is a little box with cards. There are words on each card. Pick a card, read the word(s) on the card and tell me what you think.		
□ Acceptance□ Activities at school□ Activities by school□ Adjustments□ Applying for a job□ Assignments□ Assistive devices/tools□ Break□ Cafeteria□ Choosing for yourself□ Class□ Clothing/shoes□ Colleagues□ Competitions□ Chores□ Doctor□ Equality	□ Friends□ Physiotherapy□ Gym class□ Health□ Home□ Hospital□ Independence□ Individual□ Free hours□ Leisure□ Lessons/classes□ Mentor□ Money□ Moving about at school□ Operation□ Parents□ Peers with disability	□ Rehabilitation□ Schoolyard□ Self‐confidence□ Job□ Sports field□ Students□ Taking care of yourself□ Teacher□ Team mates□ Testing□ Therapy□ Toilet□ Trainer□ Treatment□ Work

**Table:**

Rounding off	
□ Anything else	Are there any other things you would like to share?Are there other things that you think are important that we have not discussed yet?
□ Thank you	Thank you for your willingness to participate in this interview.

## APPENDIX B

### B.1

Members of the PERRIN‐PiP Study Group: M. Ketelaar, D.W. Smits, J.M. Voorman (University Medical Center Utrecht and De Hoogstraat Rehabilitation, Utrecht), A.J. Dallmeijer (VU University Medical Center, Amsterdam), M.E. Roebroeck (Erasmus MC, University Medical Center and Rijndam Rehabilitation, Rotterdam), H.A. Reinders‐Messelink (Revalidatie Friesland and University Medical Center Groningen), J.W. Gorter (McMaster University, Hamilton, Canada), J. Verheijden, BOSK (Association of Physically Disabled Persons).
